# First bite syndrome following surgical management of primary jugular foramen lesion: Clinical experience and review of the literature

**DOI:** 10.1016/j.bjorl.2025.101629

**Published:** 2025-05-03

**Authors:** Xinrui Xu, Mengye Ma, Qianru Wu, Xunbei Shi, Feitian Li, Chunfu Dai

**Affiliations:** a Fudan University, Eye, Ear, Nose, and Throat Hospital, Department of Otology and Skull Base Surgery, Shanghai, China; b Fudan University, Eye, Ear, Nose, and Throat Hospital, Ministry of Health, Key Laboratory of Hearing Medicine, Shanghai, China

**Keywords:** First bite syndrome, Jugular foramen lesion, Sympathetic plexus, Internal carotid artery, Surgical management

## Abstract

**Objective:**

This study presents patients with primary jugular foramen lesions who developed First Bite Syndrome (FBS) after surgery, and reviews the literature on advancements in treating FBS.

**Methods:**

A retrospective review of 47 patients with primary jugular foramen lesions who underwent surgery between January 2020 and May 2022 was conducted.

**Results:**

FBS were identified in 4 patients (8.5%). All 4 patients underwent Infratemporal Fossa (ITF) dissection, Internal Jugular Vein (IJV) ligation, and Internal Carotid Artery (ICA) dissection. None of the patients had the External Carotid Artery (ECA) ligated. The mean duration of FBS was 16-months (range, 6–25 months) with a mean follow-up time of 22 months (range, 20–26 months). All patients reported tolerable FBS symptoms, with an average pain intensity of 2.8 (range, 2–4). None received medical treatment.

**Conclusion:**

Patients with primary jugular foramen lesion who undergo ICA dissection may be at risk for developing FBS. However, symptoms are usually mild, and a conservative, wait-and-see strategy is recommended. All patients undergoing surgery for jugular foramen lesions should be informed about the potential risk of postoperative FBS.

**Level of evidence:**

4.

## Highlights

• First bite syndrome may occur after resection of primary jugular foramen lesions.
• Symptoms of first bite syndrome is mild in these patients postoperatively.
• The rationale could be push of the sympathetic plexus along the internal carotid artery.

## Introduction

First Bite Syndrome (FBS) is described as severe sharp or cramping facial pain in the parotid region that occurs with initial bite of a meal and typically improves after several subsequent bites. The condition was first described by Netterville et al. in 1998. In their study, eight out of nine patients with FBS underwent sympathetic trunk resection, while the remaining patient presented with sympathetic dysfunction, manifesting as Horner syndrome. Based on these findings, Netterville et al. hypothesized that FBS results from parasympathetic hyperactivation of the myoepithelial cells within the parotid gland.^1^

Linkov et al. identified Parapharyngeal Space (PPS) dissection, deep lobe of parotid resection, and sympathetic chain sacrifice as the strongest independent risk factors for the development of postoperative FBS.^2^ Previous studies on FBS have primarily focused on PPS and parotid gland surgery.^3^, ^4^, ^5^ However, FBS following resection of tumors originated from the jugular foramen has not been reported yet. In an attempt to better understand this rare syndrome, we present a series of cases of FBS following surgical management of jugular foramen lesions. The objective of this study is to investigate the incidence and potential risk factors associated with postoperative FBS in patients undergoing resection of primary jugular foramen lesions.

## Methods

The medical ethics committee of Eye, Ear, Nose and Throat Hospital of Fudan University approved the study. A total of 47 patients who underwent surgical resection of jugular foramen lesions at our institution during January 2020 to May 2022 were retrospectively reviewed. Data on demographics, clinical characteristics, and details of surgical procedures were retrieved. Patients were divided into FBS group (FBS+) and non-FBS group (FBS-) according to whether there was postoperative development of FBS. The intensity of FBS symptoms was evaluated using a Visual Analog Pain scale (VAS) of 1–10, with 1 indicating mild pain that can be ignored and 10 representing the worst possible pain during each meal. All patients were followed up by telephone and outpatient visit postoperatively.

## Results

The demographic and clinical characteristics of the patients are shown in Table 1. A total of four patients with FBS were identified, representing 8.5% of all patients who underwent surgical resection of jugular foramen lesions (4 out of 47). The FBS group consisted of three men and one woman, with a mean age of 46 years (range: 33–53 years) at the time of symptom onset. The mean age of the non-FBS group was 45 years (range 17–76 years). Surgical details are outlined in Table 2. FBS was associated with two histopathological diagnoses in the present study: chondrosarcoma (1/4) and paraganglioma (3/4). Gross total tumor removal was achieved in all patients. All four patients with FBS underwent type A Infratemporal Fossa (ITF) dissection, ligation of the Internal Jugular Vein (IJV), and dissection of the internal carotid artery (ICA). Among the 43 patients without FBS, ITF type A dissection was performed in all, ICA dissection in 10, and IJV ligation in 37. External Carotid Artery (ECA) ligation was not performed in either group. Follow-up data are shown in Table 3. FBS appeared postoperatively in the second week in three patients and in the sixth week in one patient. The mean duration of FBS was 16 months (range, 6–25 months). Pain episodes lasts between 3–10 seconds during eating. The patients characterized the pain as cramp (1/4), sharp (1/4) or dull (2/4), with radiation to the ear (4/4). The average intensity of symptoms on the pain scale (1–10) was 2.8 (range: 2–4). In three patients, pain was worsened by sour or cold foods. Additionally, three patients reported modifications in eating behavior to ease pain, such as chewing on the other side, taking smaller bites, rubbing the parotid region, and eating slowly. None of the patients received specific treatment for FBS. At the last follow-up, two patients reported complete resolution of symptoms, one reported partial resolution, and one patient continued to experience persistent FBS. None of the patients developed Frey’s or Horner syndrome.

**Table 1:** Patient demographics and clinical characteristics.

Characteristics	FBS- (n = 43)	FBS+ (n = 4)
Age (years)		
Mean	45	46
Range	17–76	33–53
Sex (Male/Female)		
Female	28 (96.6%)	1 (3.4%)
Male	15 (83.3%)	3 (16.7%)
Pathology		
Chondrosarcoma	9 (90.0%)	1 (10.0%)
Jugular paraganglioma	23 (88.5%)	3 (11.5%)
Schwannoma	6 (100%)	0 (0%)
Meningioma	5 (100%)	0 (0%)
ITF dissection		
Yes	43 (91.5%)	4 (8.5%)
No	0 (0%)	0 (0%)
ECA ligation		
Yes	0 (100%)	0 (0%)
No	43 (91.5%)	4 (8.5%)
ICA dissection		
Yes	10 (71.4%)	4 (28.6%)
No	33 (100%)	0 (0%)
IJV ligation		
Yes	37 (90.2%)	4 (9.8%)
No	6 (100%)	0 (0%)

ITF, Infratemporal Fossa; ECA, External Carotid Artery; ICA, Internal Carotid Artery; IJV, Internal Jugular Vein.

**Table 2:** Surgical details of patients with FBS.

Case Nº	Age	Sex	Pathology	ITF dissection	ECA ligation	ICA dissection	IJV ligation
1	47	M	Chondrosarcoma	Yes	No	Yes	Yes
2	53	F	Malignant PGL	Yes	No	Yes	Yes
3	50	M	PGL	Yes	No	Yes	Yes
4	33	M	PGL	Yes	No	Yes	Yes

PGL, Paraganglioma; ITF, Infratemporal Fossa; ECA, External Carotid Artery; ICA, Internal Carotid Artery; IJV, Internal Jugular Vein.

**Table 3:** Follow-up data on pain.

Case Nº	Time from surgery to FBS (weeks)	Duration per attack (seconds)	Duration of FBS (months)	Treatment for FBS	Type of pain	Radiation of pain	Intensity of pain	Improvement of pain	Food worsens the pain	Modification of eating behavior
1	2	5	16	None	Cramp	To the ear	3	Resolution	None	Chew on other side, small bites, rub on the parotid region
2	6	3	6	None	Sharp	To the ear	2	Resolution	Sour food	No
3	2	3	25	None	Dull	To the ear	2	Partial	Sour or cold food	Chew on other side, eat slowly
4	2	10	18	None	Dull	To the ear	4	No	Sour food	Rub on the parotid region

Patient 1 was a 47-year-old male with primary chondrosarcoma in the left jugular foramen. He presented with progressive facial palsy for one year, along with hoarseness and cough for the past two months. The patient underwent tumor resection via the ITF type A approach, with a facial nerve graft. Intraoperative findings revealed that the tumor had penetrated the PPS and the ICA. FBS developed two weeks postoperatively. The pain lasts 16 months and resolved following a second surgery. The patient did not receive any treatment for FBS, as the symptoms were deemed tolerable.

Patient 2 was a 53-year-old woman with a right-sided C2 jugular paraganglioma. She complained of pulsatile tinnitus and hearing loss for two years. The patient underwent resection using an ITF type A approach, with tension-free anterior rerouting of the facial nerve. FBS symptoms developed 6 weeks postoperatively. The pain persisted for 6 months and subsequently resolved completely without the need for medical treatment.

Patient 3 was a 50-year-old male with a malignant paraganglioma in the right jugular foramen. He had a history of facial palsy and hearing loss for over 20 years. The patient underwent resection via an ITF type A approach. Intraoperative findings revealed that the tumor had infiltrated the ICA, resulting in unavoidable tumor residue. FBS symptoms developed two weeks postoperatively. The pain persisted for 25 months and was partially resolved at the time of the last follow-up. He also did not receive medical intervention to control his FBS symptoms.

Patient 4 was a 33-year-old male with a right-sided C2 jugular paraganglioma. He presented hearing loss and tinnitus for half a year. This patient underwent resection via an ITF type A approach, with partial tension-free anterior rerouting of the facial nerve. FBS symptoms developed two weeks postoperatively. The patient described moderate pain that interfered with daily activities, but he did not seek medical treatment due to the tolerable nature of the pain and concerns about potential side effects. The FBS persisted without improvement for 18 months till now.

## Discussion

### Clinical characteristics

Jugular foramen tumors are rare disorders that can affect vital adjacent neurovascular structures. Most otoneurologists prefer the ITF approach for tumor resection in the jugular foramen. Previous studies demonstrated that FBS is a potential complication following surgical removal of tumors involving the ITF. However, Linkov et al. reported an inverse association between ITF dissection and postoperative FBS development (OR = 0.15; 95% CI 0.04‒0.63; *p* = 0.003). In the present study, all patients underwent tumor removal using the ITF approach. Of the 47 patients, 4 (8.5%) patients suffered from FBS postoperatively, compared to the 2%–30% incidence of FBS reported after parotidectomy and upper neck surgery in the literature.^2^, ^6^, ^7^ In contrast to the female predominance observed in previous studies, 3 out of 4 patients with FBS in our series were male. This difference may be attributed to the small sample size in our study. The duration of symptoms associated with postoperative FBS may vary from several days to months.^3^, ^7^ In our study, the onset of FBS symptoms occurred between 2–6 weeks postoperatively. Episodes of FBS pain have been reported to last from seconds to a few minutes in the literature.^5^ In our study, all patients described FBS pain episodes lasting between 3 and 10 seconds, which is consistent with previous findings. Abdeldaoui et al. identified acidic food as a trigger factor for FBS in their cohort,^8^ which was similarly observed in our study, where pain was exacerbated by sour or cold foods in 3 patients. The duration of FBS pain in the literature was highly varied. Chiu et al. followed 10 patients after PPS surgery for a mean time of 21 months and found that 8 patients experienced persist FBS, while 1 patient had spontaneous resolution.^3^ Likewise, after reviewing 17 patients after upper cervical surgery with a mean follow-up of 47 months, Abdeldaoui et al. reported persist FBS in 15 patients, with 3 experiencing spontaneous resolution.^8^ However, Xu et al., observed spontaneous resolution of FBS in 7 out of 8 patients after parotidectomy, with follow-up ranging from 3.3 to 39.5 months.^6^ We postulated that the cervical sympathetic trunk was sacrificed in a large proportion of patients in the studies by Chiu et al. and Abdeldaoui et al. may contribute to the discrepancy in FBS duration. The average intensity of pain evaluated by the pain scale was 8.5 and 5.7 in the studies by Abdeldaoui et al. and Linkov et al., respectively.^2^, ^8^ In these studies, most patients with FBS received medical treatment due to the severity of pain. In contrast, the average pain intensity in our study was 2.8, and none of the patients required medical intervention, as the FBS pain was tolerable. Taken together, the characteristics of shorter pain duration and lower intensity in our series suggest that FBS is generally mild in the context of resection of jugular foramen lesions using the ITF approach.

### Mechanism

Table 4 summarizes the literature on postoperative FBS (with >3 cases per study), most of which report FBS following PPS or parotid gland surgery. The prevailing mechanism for postoperative FBS is the sympathetic denervation theory, proposed by Netterville et al., which suggests that loss of sympathetic innervation to the parotid gland leads to hypersensitivity of sympathetic receptors on myoepithelial cells. The subsequent parasympathetic release of neurotransmitter acetylcholine cross-stimulated these hypersensitive sympathetic receptors, causing an exaggerated contraction of the myoepithelial cells and FBS pain. This theory is supported by case series in which the cervical sympathetic trunk was resected in almost half of the patients, and others exhibited sympathetic dysfunction or underwent External Carotid Artery (ECA) ligation.^3^, ^8^ The cervical trunk contains three interconnected ganglions: the superior, middle, and inferior cervical ganglion. The Superior Cervical Ganglion (SCG) lies approximately at the levels of the second and third cervical vertebra, posterior to the carotid space, lateral to the longus colli muscle, and anteromedial or medial to the ICA. Postganglionic sympathetic fibers leave the SCG, then travel along the ECA and ICA as a nerve plexus to innervate targets in the head and neck. The ECA nerve plexus further travels along its branch vessels, such as the meningeal artery, then passes through the otic ganglion to supply the parotid gland. Thus, surgical disruption of any part of the sympathetic pathway to the parotid gland could result in FBS. This may explain why FBS can occur in the absence of Horner syndrome. FBS has also been described in patients without surgery, who suffered from malignancies in the submandibular gland, parotid gland, or parapharyngeal space, it is defined as primary FBS.^9^, ^10^, ^11^, ^12^ In the primary FBS, the sympathetic plexus to the parotid gland may be directly invaded by tumors located in the parotid gland and parapharyngeal space or indirectly by submandibular gland malignancies through perineural infiltration.

**Table 4:** Postoperative first bite syndrome reported in the literature (n>3).

References	Year	Nº of cases	Pathology	Surgical procedure	Treatment	Relief
Netterville et al.	1998	9	Vagal paraganglioma (9)	Sympathetic trunk resection (8) NA (1)	Removal of the auriculotemporal nerve (1)	Yes
Chiu et al.	2002	12	Sympathetic chain neuroma (4) Paraganglioma (3) DLP pleomorphic adenoma (3)	Sympathetic chain resection (5) ECA ligation (5)	Nonsteroidal anti-inflammatory medications (6) Carbamazepine (1) Tympanic neurectomy (3) Spontaneous resolution (2)	No
Kawashima et al.	2008	9	Sympathetic chain Schwannomma (3) DLP pleomorphic adenoma (3) Unknown origin pleomorphic adenoma (3)	Sympathetic chain resection (3) ECA ligation (1) DLP resection (6)	NA	NA
Lee et al.	2009	5	Carotid body tumor (2) DLP pleomorphic adenoma (1) Sympathetic chain schwannomma (1) Retropharyngeal lymph node metastatic papillary carcinoma (1)	NA	Local injection of botulinum toxin type A (5)	Partial
Linkov et al.	2012	45	Schwannomma (10) Paraganglioma (9) Pleomorphic Adenoma (20) NA (6)	Sympathetic chain resection (9) ECA ligation (6) DLP resection (25) PPS dissection (42) ITF dissection (2)	Algesic (9) Opioid (5) Neuropathic pain medication (10) Acupuncture (1) Botox injection (1)	NA
Abdeldaoui et al.	2013	17	Paraganglioma (8) Vagal or sympathetic schwannoma (5) Pleomorphic adenoma (3) Warthin's tumour (1)	Sympathetic chain resection (10) ECA ligation (7) DLP resection (2)	Non-steroidal anti-inflammatory drugs combined with anticonvulsants or calcium channel blockers or tricyclic antidepressants (14) No treatment (3)	Partial
Ghosh et al.	2016	5	Carotid body tumor (2) Parotid pleomorphic adenoma (2) Temporomandibular joint giant cell tumor (1)	PPS dissection (5)	Intraparotid injection of botulinum toxin type A (5)	Partial
Avinçsal et al.	2017	16	Pleomorphic adenoma (9) Schwannoma (3) Paraganglioma (2) NA (2)	Sympathetic chain resection (3) ECA ligation (12) PPS dissection (10)	Spontaneous resolution (8) NA (8)	No
Topf et al.	2018	6	Oropharyngeal squamous cell carcinoma: Tonsil (4) Base of tongue (1) Tonsil& Base of tongue (1)	Transoral robotic surgery (6) Ligation of at least two ECA vessels (6)	Pregabalin, TheraBite (1) Gabapentin, Botox (1) Opioids (1) No treatment (3)	Yes
Lammek et al.	2021	7	Parotid pleomorphic adenoma (6) Warthin’s tumour (1)	NA	Spontaneous resolution (6) NA (1)	Yes
Xu et al.	2022	8	Parotid carcinoma ex pleomorphic adenoma (2) Parotid pleomorphic adenoma (4) Acinic cell carcinoma (1) Parotid pleomorphic adenoma, adenoid cystic carcinoma (1)	DLP resection (6) Superficial lobe parotidectomy (2)	Spontaneous resolution (7) Gabapentin (1)	Yes
Wistermayer et al.	2024	7	Palatine tonsil squamous cell carcinoma (7)	Dissection of parapharyngeal fat (7)	NA	Yes

DLP, Deep Lobe Parotid; ITF, Infratemporal Fossa; ECA, External Carotid Artery; PPS, Parapharyngeal Space; NA, Not Available.

**Fig. 1:**
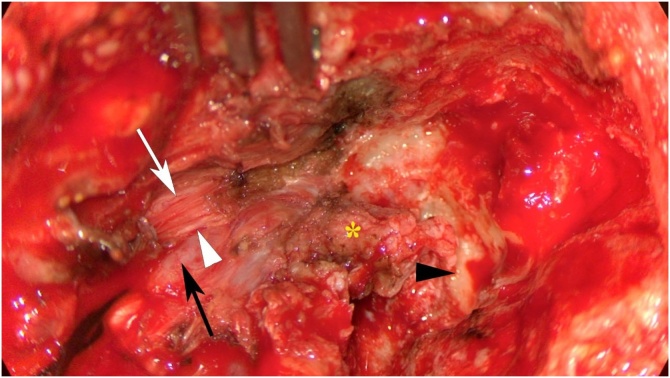
Intraoperative photograph revealed the exposure of the tumor (yellow asterisk), promontory (black arrowhead), ligation of the internal jugular vein (black arrow), internal carotid artery (white arrow), and post-ganglion sympathetic fiber along the internal carotid artery.

Topf et al. observed that in patients received transoral resection of the PPS tumor but did not have transcervical arterial ligation, none developed FBS; therefore, they suggested that vessel ligation, rather than dissection in PPS, was a significant risk factor for FBS following PPS surgery.^13^, ^14^ While Wistermayer et al. found that manipulation of PPS fat, rather than ligation of ECA vessels, was a risk factor for developing FBS. These discrepancy may due to different surgical technique during handle PPS and carotid artery vessels.^15^ ITF dissection used to be associated with FBS due to possible exposure of the meningeal artery. In our study, all patients with FBS underwent type A ITF dissection; thus the meningeal artery was intact in these patients. ICA dissection and IJV ligation were performed in the 4 FBS patients. In addition, paraganglioma in the jugular foramen has a tendency to grow inferior to affect the ICA. Taken together, we postulated that the sympathetic plexus may be accidentally pushed during ICA manipulation or IJV ligation, leading to the mild FBS symptoms observed in our series (Fig. 1).

### Management

Currently, there is no consensus on the treatment of FBS, with management strategies varying depend on the pain intensity. In our study, all FBS patients experienced mild pain, which partially resolved in one case; therefore, observation is an appropriate treatment option. However, in patients with debilitating FBS pain, pharmacological treatment, surgical intervention, and radiotherapy should be undertaken for patient consultation. Pharmacological treatments include Non-Steroidal Anti-Inflammatory Drugs (NSAIDs), neuropathic agents, and botulinum toxin injection into the parotid region. However, NSAIDs were found to be ineffective in several case reports.^3^, ^16^, ^17^ Neuropathic agents, including calcium channel blockers such as gabapentin and pregabalin, as well as anticonvulsants like carbamazepine, have been shown to reduce pain intensity in the majority of patients, although some individuals may not respond.^3^, ^8^, ^11^, ^13^ Tricyclic antidepressants, such as amitriptyline, have also been reported to yield variable responses.^8^, ^18^

### Botulinum toxin

Botulinum toxin injected into the parotid gland had been shown to be an effective treatment for alleviating FBS symptoms. Botulinum toxin works by inhibiting the release of acetylcholine at the presynaptic terminal of the injection of 75 units of botulinum toxin into the parotid region, targeting areas of the most intense pain reported by the patient, and achieved complete symptom relief over a 10-weeks follow-up.^19^ Since then, numerous studies have evaluated the efficacy of botulinum toxin injections in FBS, utilizing different dosages and injection techniques. Lee et al. presented 5 FBS patients treated with a lower dose of botulinum toxin (33 units fractionated into 3 doses of 11 units each) injected into the parotid region. Pain relief was achieved within 3–4 months, but after 6 months, the pain recurred with the same intensity in four patients, and with mild improvement in one.^20^ Similarly, Sims et al. found that two of three FBS patients experienced complete resolution of symptoms after receiving a 75-unit botulinum toxin injection, while the third patient achieved near-complete resolution. However, symptoms recurred within 3–5 months, necessitating repeat injections every 3–8 months.^21^ Ghosh et al. reported significant improvement in three of four FBS patients following multisite botulinum toxin injections, with doses ranging from 17.5 to 50 units. Although complete relief was not achieved, symptoms recurred with initial severity around 4 months after injection, prompting one to four repeat injections every 4 months. The duration before complete symptom resolution ranged from 10 to 28 months after the first injection.^22^ Notably, no complications, including facial paralysis, infection, or allergic reactions, have been reported in these studies. In summary, while botulinum toxin appears to be a promising and safe treatment for FBS, the optimal dosage, injection strategy, timing of pain relief, and long-term safety still require further investigation.

### Surgery

The inferior salivary nucleus sends preganglionic fibers through the glossopharyngeal nerve and tympanic plexus to the otic ganglion, then innervates the parotid gland with parasympathetic fibers. Based on this neuroanatomical pathway, tympanic neurectomy was used to treat FBS. Chiu et al. reported that three patients who underwent tympanic neurectomy experienced minimal improvement, with two patients reporting only slight relief for one week, and the third patient experiencing no benefit from the procedure.^3^ Ali et al. presented a patient underwent tympanic neurectomy achieved only 50% symptom relief. A second tympanic neurectomy combined with laser ablation of the promontory was performed six months later, but no further improvement was observed.^19^ Amin et al. also described a FBS case alleviated by CO_2_ laser ablation of the tympanic plexus.^23^ The exact effect of tympanic neurectomy in FBS treatment needs to be further discussed.

### Radiotherapy

Chiu et al. first described a patient whose FBS symptoms resolved 7 months after onset while receiving radiation therapy.^3^ Costa et al. later reported two patients who developed FBS following major cervical oncologic surgeries and did not respond to NSAIDs. However, both patients had complete FBS resolution after subjected to adjuvant radiotherapy, with asymptomatic periods of 7- and 10-months during follow-up, respectively.^17^ Previous animal studies have demonstrated that radiotherapy reduced alpha-Smooth Muscle Actin (α-SMA) and myofilaments in myoepithelial cells, directly altering the function of salivary glands.^24^, ^25^ These findings suggested that radiotherapy alleviates FBS by affecting the myoepithelial cells of the parotid gland. Additionally, stereotactic radiosurgery has been shown to be effective in treating chronic intractable pain with acceptable complications. However, with the advent of neuromodulation and intrathecal opioid therapies, radiotherapy has gradually been abandoned in pain treatment.^26^ In summary, radiotherapy could be an option for patients with severe FBS that is resistant to other medical treatments. However, patients should be informed of the potential adverse effects of radiotherapy before its application. The limitations of our study include the small sample size and the inherent bias of retrospective design. Future prospective studies are needed to gain a more accurate understanding of this etiology.To the best of our knowledge, no cases of postoperative FBS have been reported in patients with primary jugular foramen lesions. This is the first study to analyze risk factors for postoperative FBS in patients with primary jugular foramen lesions.

## Conclusion

FBS can occur following the resection of primary jugular forman lesions; however, postoperative FBS symptoms are typically mild and do not require medical intervention. The possible cause of FBS in these patients is inadvertent manipulation of the sympathetic plexus during ICA management. Surgeons should counsel patients regarding the potential risk of FBS prior to surgery and remain vigilant in identifying this rare condition postoperatively.

## Funding

This work was sponsored by Shanghai Municipal key clinical specialty (shslczdzk00801), Natural Science Foundation (nº 82201289).

## Declaration of competing interest

The authors declare that there is no funding, financial relationships, or conflicts of interest to disclose regarding the publication of this paper.

## References

[1] Netterville J.L., Jackson C.G., Miller F.R., Wanamaker J.R., Glasscock M.E. (1998). Vagal paraganglioma: a review of 46 patients treated during a 20-year period. Arch Otolaryngol Head Neck Surg..

[2] Linkov G., Morris L.G., Shah J.P., Kraus D.H. (2012). First bite syndrome: incidence, risk factors, treatment, and outcomes. Laryngoscope..

[3] Chiu A.G., Cohen J.I., Burningham A.R., Andersen P.E., Davidson B.J. (2002). First bite syndrome: a complication of surgery involving the parapharyngeal space. Head Neck..

[4] Kawashima Y., Sumi T., Sugimoto T., Kishimoto S. (2008). First-bite syndrome: a review of 29 patients with parapharyngeal space tumor. Auris Nasus Larynx..

[5] Lammek K., Tretiakow D., Skorek A. (2021). First bite syndrome after parotidectomy: a single-centre experience. Int J Oral Maxillofac Surg..

[6] Xu V., Gill K.S., Goldfarb J., Bovenzi C., Moayer R., Krein H. (2022). First bite syndrome after parotidectomy: a case series and review of literature. Ear Nose Throat J..

[7] Avinçsal M.Ö, Hiroshima Y., Shinomiya H., Shinomiya H., Otsuki N., Nibu K.I. (2017). First bite syndrome - An 11-year experience. Auris Nasus Larynx..

[8] Abdeldaoui A., Oker N., Duet M., Cunin G., Tran Ba Huy P. (2013). First Bite Syndrome: a little-known complication of upper cervical surgery. Eur Ann Otorhinolaryngol Head Neck Dis..

[9] Guss J., Ashton-Sager A.L., Fong B.P. (2013). First bite syndrome caused by adenoid cystic carcinoma of the submandibular gland. Laryngoscope..

[10] Diercks G.R., Rosow D.E., Prasad M., Kuhel W.I. (2011). A case of preoperative “first-bite syndrome” associated with mucoepidermoid carcinoma of the parotid gland. Laryngoscope..

[11] Masood M.M., Giosia M.D., Hackman T.G. (2018). Chronic atypical first bite syndrome and primary squamous cell carcinoma of the parotid. Head Neck..

[12] Lieberman S.M., Har-El G. (2011). First bite syndrome as a presenting symptom of a parapharyngeal space malignancy. Head Neck..

[13] Topf M.C., Moritz E., Gleysteen J., Curry J.M., Cognetti D.M., Luginbuhl A.J. (2018). First bite syndrome following transcervical arterial ligation after transoral robotic surgery. Laryngoscope..

[14] Boyce B.J., Curry J.M., Luginbuhl A., Cognetti D.M. (2016). Transoral robotic approach to parapharyngeal space tumors: case series and technical limitations. Laryngoscope..

[15] Wistermayer P.R., Brown A.E., Cave T.B., Klusovsky L.E., Chang B.A., Hayden R.E. (2024). First bite syndrome in transoral surgery for oropharyngeal cancer. Otolaryngol Head Neck Surg..

[16] Phillips T.J., Farquhar-Smith W.P. (2009). Pharmacological treatment of a patient with first-bite syndrome. Anaesthesia.

[17] Costa T.P., de Araujo C.E.N., Filipe J., Pereira A.M. (2011). First-bite syndrome in oncologic patients. Eur Arch Otorhinolaryngol..

[18] Albasri H., Eley K.A., Saeed N.R. (2011). Chronic pain related to first bite syndrome: report of two cases. Br J Oral Maxillofac Surg..

[19] Ali M.J., Orloff L.A., Lustig L.R., Eisele D.W. (2008). Botulinum toxin in the treatment of first bite syndrome. Otolaryngol Head Neck Surg..

[20] Lee B.J., Lee J.C., Lee Y.O., Wang S.G., Kim H.J. (2009). Novel treatment of first bite syndrome using botulinum toxin type A. Head Neck..

[21] Sims J.R., Suen J.Y. (2013). First bite syndrome: case report of 3 patients treated with botulinum toxin and review of other treatment modalities. Head Neck.

[22] Ghosh A., Mirza N. (2016). First bite syndrome: our experience with intraparotid injections with botulinum toxin type A. Laryngoscope..

[23] Amin N., Pelser A., Weighill J. (2014). First bite syndrome: our experience of laser tympanic plexus ablation. J Laryngol Otol..

[24] Hakim S.G., Kosmehl H., Lauer I., Nadrowitz R., Wedel T., Sieg P. (2002). The role of myoepithelial cells in the short-term radiogenic impairment of salivary glands. An immunohistochemical, ultrastructural and scintigraphic study. Anticancer Res..

[25] Hakim S.G., Schroder C., Geerling G., Lauer I., Wedel T., Kosmehl H. (2006). Early and late immunohistochemical and ultrastructural changes associated with functional impairment of the lachrymal gland following external beam radiation. Int J Exp Pathol..

[26] Roberts D.G., Pouratian N. (2017). Stereotactic radiosurgery for the treatment of chronic intractable pain: a systematic review. Oper Neurosurg (Hagerstown)..

